# The Combination Therapy With Sorafenib in Therapeutic Strategy of Acute Myeloid Leukemia. Is It Promising? A Narrative Review

**DOI:** 10.1002/hsr2.71412

**Published:** 2025-10-24

**Authors:** Mobina Nakhaei Shamahmood, Abolfazl Miri, Fatemeh Mezginejad

**Affiliations:** ^1^ Student Research Committee Birjand University of Medical Sciences Birjand Iran; ^2^ Student Research Committee Zahedan University of Medical Sciences Zahedan Iran; ^3^ Department of Hematology, School of Allied Medicine, Cellular and Molecular Research Center Birjand University of Medical Sciences Birjand Iran

**Keywords:** AML, combination, sorafenib

## Abstract

**Background and Aims:**

Acute myeloid leukemia (AML) is the most common acute leukemia, which is associated with high morbidity and mortality, and its incidence rate is increasing. Despite tremendous advancements in AML treatment, managing the condition is still challenging. Receptor kinase inhibitors like sorafenib have recently raised concerns about monotherapy, and combination treatments are now being investigated as a potential therapeutic avenue. To classify medications taken in conjunction with sorafenib and analyze their combined effects, we reviewed the prior research findings.

**Methods:**

A thorough literature search utilizing several databases, including PubMed, Google Scholar, Scopus, Embase, and Cochrane, yielded 78 relevant articles until October 2023. Search terms including AML, combination, and Sorafenib were utilized during the database search to find relevant papers.

**Results:**

Complete remission (CR) in the combination of sorafenib with “cytarabine and idarubicin”, “Vorinostat and Bortezomib, Clofarabine”, “Fludarabine and Busulfan”, “5‐azacytidine” and “Quizartinib, Midostaurin and Giltertinib” respectively “99% and 94%”, “27% and 3%”, 66.6%, “45%, 26%, 16%”, “71% and 52%”. Furthermore, the overall survival (OS) values for the combination of sorafenib with “Quizartinib, Midostaurin, and Giltertinib”, “cytarabine, daunorubicin” and “5‐azacitidine” were “80%” “62% 45%”, and 24%, respectively.

**Conclusion:**

Accordingly, several studies suggest that sorafenib's anticancer effectiveness may be enhanced with chemotherapy drugs.

## 1 Introduction

Acute myeloid leukemia (AML) is the most prevalent acute leukemia, which is expected to occur in 3 cases for every 100,000 individuals [1, 2, 3, 4]. Based on cytogenetic and molecular characteristics, the prognosis varies significantly for this clonal disease, which is a heterogeneous hematological malignancy with high morbidity and mortality. The disease is derived from abnormally and occasionally poorly differentiated hematopoietic system cells [5, 6, 7]. An estimated 11,000 new AML patients are diagnosed annually, with a median age at presentation of 65 years, and regrettably, the incidence rate is rising [8]. Therefore, efforts to find a novel, prospective, and successful treatment plan are ongoing, and in doing so, consideration is given to both drug repositioning and the discovery of new drugs.

The oral bi‐aryl urea Sorafenib (Nexavar, Bayer) is a small molecule inhibitor of RAF serine/threonine kinase isoforms that are known to regulate the Raf kinase/mitogen‐activated Erk kinase/extracellular signal‐regulated kinase (RAF/MEK/ERK) pathway [9, 10]. Moreover, vascular endothelial growth factor receptor (VEGFR), platelet‐derived growth factor receptor (PDGFR), fibroblast growth factor receptor (FGFR), FMS‐like tyrosine kinase 3 (FLT3), c‐KIT, and RET proteins were demonstrated to be inhibited by sorafenib. Therefore, it is believed that sorafenib's anticancer efficacy stems from its capacity to suppress tumor development and damage tumor microvasculature via antiproliferative, antiangiogenic, and or proapoptotic effects. Specifically, sorafenib attaches itself to target kinases in their inactive conformation at the adenosine 5′‐triphosphate (ATP) site [11, 12]. At present, Sorafenib is authorized by the European Medicines Agency to treat patients with advanced RCC and HCC, as well as by the US Food and Drug Administration to treat patients with unresectable HCC and advanced renal cell carcinoma (RCC). Promising results have also been observed in hepatocellular HCC. Recent phase III randomized studies in HCC have demonstrated a statistically significant increase in 3‐month survival [9].

On the other hand, the sorafenib capacity to inhibit several receptor kinases led to the initiation of preclinical and clinical research on leukemias, including AML. Effective Sorafenib antileukemic activity has been consistently shown in FLT3‐ITD positive cell lines, indicating that FLT3 activating mutations are a crucial cause of AML, increasing the risk of recurrence and decreasing overall survival [13, 14].

Although AML treatment has advanced significantly, patient management is still a major clinical challenge with limited therapeutic options, especially for older patients. Additionally, the long‐term prognosis for the majority of AML patients is unsatisfactory, necessitating the development of new therapeutic approaches.

The efficacy of sorafenib as a single agent in treating AML remains uncertain, prompting investigations into combination therapies to improve treatment outcomes. Hence, Besides traditional chemotherapy, several chemotherapy combinations are being studied to increase sorafenib's anti‐leukemic activity. These are antimetabolites (e.g., cytarabine, clofarabine, azacytidine, Fludarabine), fms‐like kinase 3 (FLT3) inhibitors (e.g., Quizartinib, giltertinib, midostaurin), proteasome inhibitor (e.g., bortezomib), alkylating agents (e.g., Busulfan), histone deacetylase inhibitor (e.g., vorinostat) and topoisomerase II inhibitor (e.g., idarubicin, danurubicin), which have been employed in clinical practices in recent years. Research has demonstrated that adding sorafenib to a chemotherapeutic medication boosts the cytotoxic activity in AML blasts [15]. Moreover, it has been discovered that a combination treatment approach involving sorafenib and cytarabine, a crucial element of AML chemotherapy, increases sorafenib's efficacy [16]. According to other research, individuals who had sorafenib in addition to idarubicin and cytarabine saw full morphological recovery but only partial platelet recovery [17].

Therefore, it may be necessary to supplement traditional chemotherapy with new medications that target leukemogenic molecules and pathways to increase its effectiveness.

**Figure 1:**
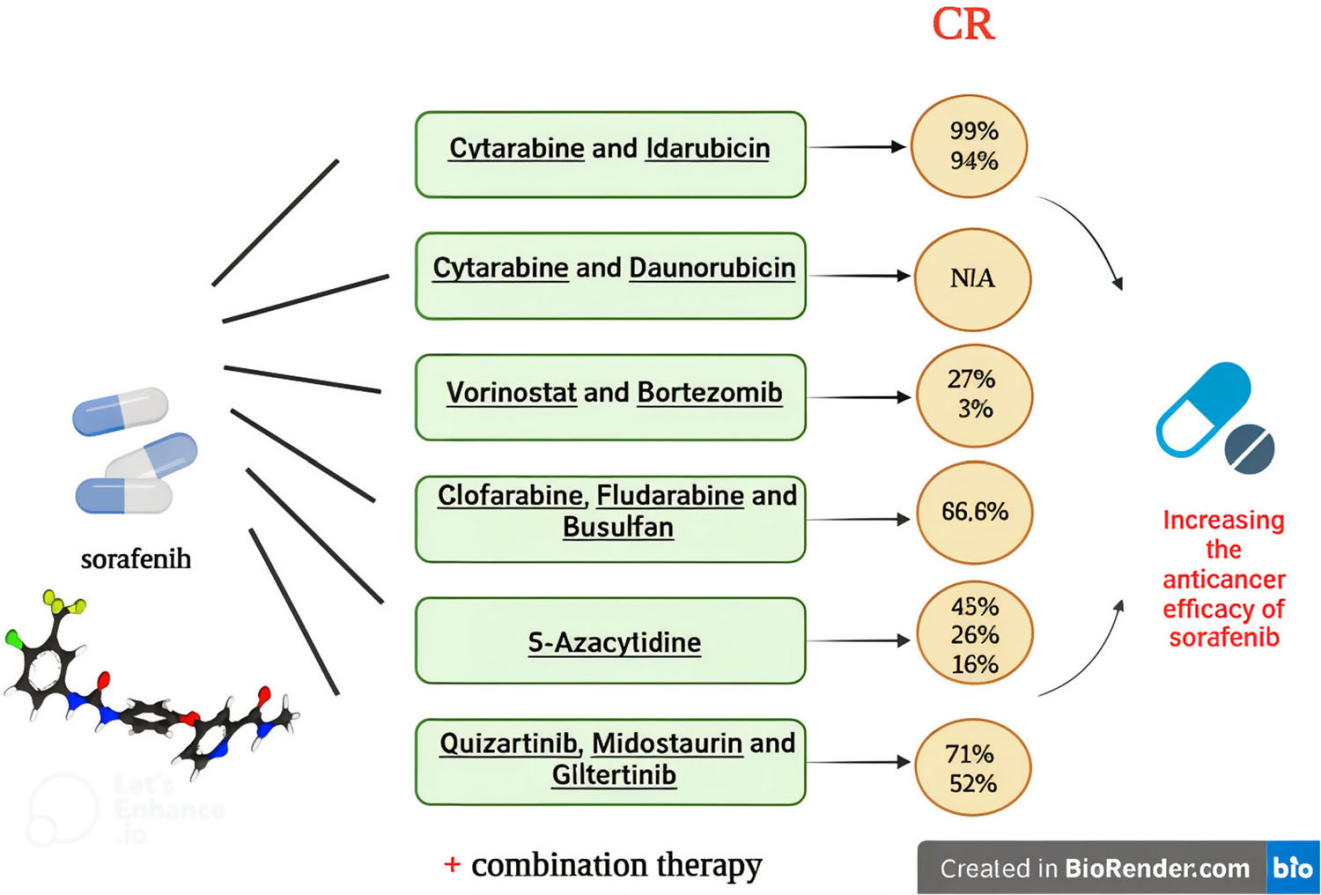
Graphical abstract.

Several diffuse investigations had been carried out in this area, but the findings varied and were not immediately available. Importantly there is not any review study focusing on the sorafenib combination therapy and gathering all these diffused studies for those working in this area. Hence, this review was done to evaluate the findings of earlier research, classify medications that are taken in conjunction with sorafenib, and illustrate and discuss their achievements (Figure 1).

## 2 Methods

**Figure 2:**
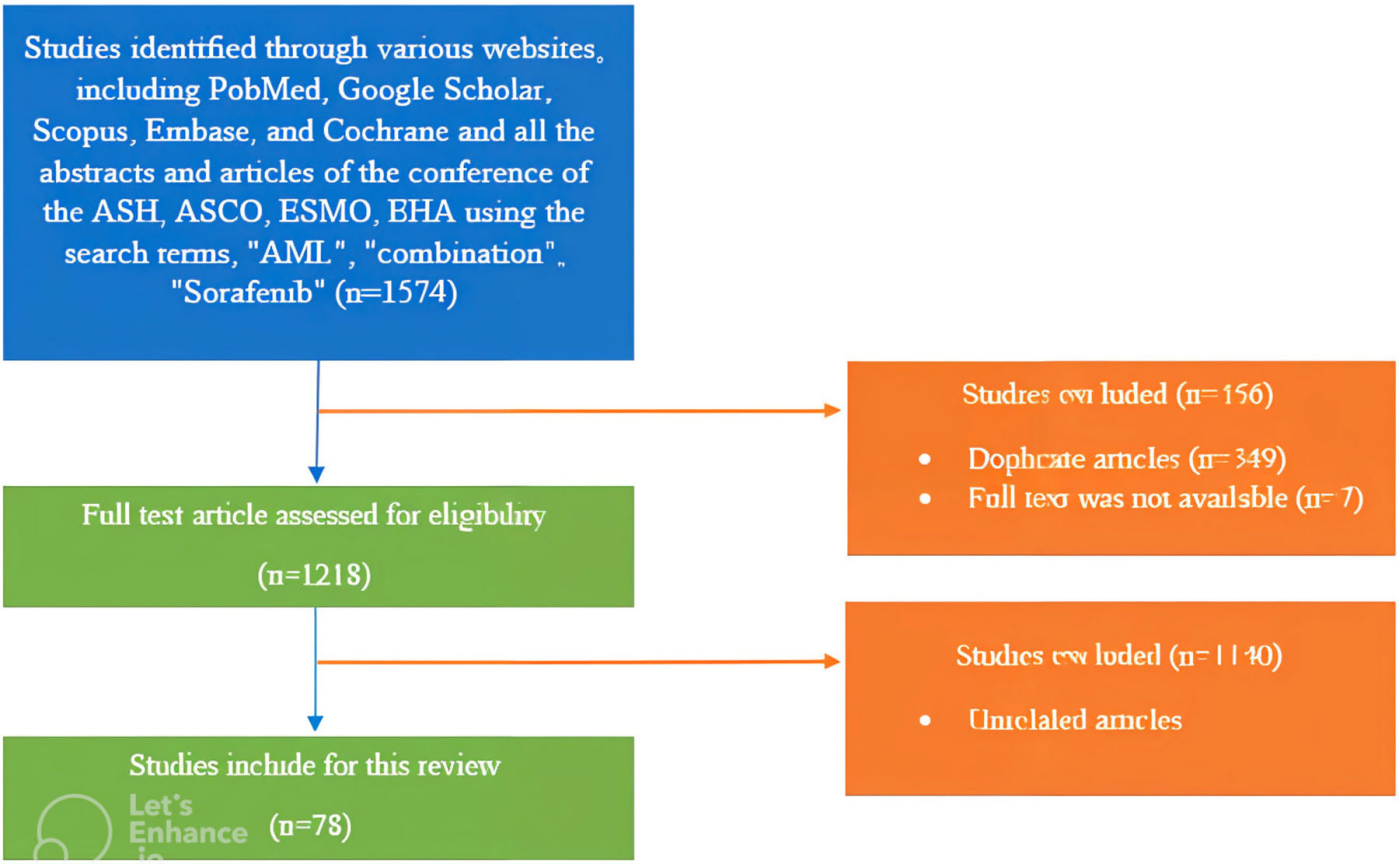
Flow diagram illustrating the methodology followed to identify the articles for the chemotherapy drugs combined with sorafenib.

Articles related to the effects of combination therapy with sorafenib in treating AML from 2007 to October 2023 were retrieved through searching databases such as PubMed, Google Scholar, Scopus, Embase, and Cochrane in English and studied for review and comparison. Also, all the abstracts and articles of the conference of the American Society of Hematology (ASH), the American Society of Medical Oncology (ASCO), Meetings, European Society of Medical Oncology (ESMO), and European Hematology Association (EHA) were reviewed. We also conducted an extensive internet search using Google to ensure no article was missed. Keywords including AML, combination, and Sorafenib were used during the database search to target similar articles. Out of a total of 1574 articles, unrelated and duplicate articles, and articles whose full text was not available, were removed and finally, 78 articles were included in the study. A flowchart of our search strategy is presented in Figure 2.

As this study is a narrative review and does not involve the collection of new human or clinical data, ethical approval and informed consent were not applicable.

### 2.1 Statistical Methods

Since this is a narrative review, no new analyses were conducted by the authors. However, where applicable, statistical results from the cited studies differentiate between pre‐specified primary analyses and exploratory or subgroup analyses, as reported in the original publications. Also the original studies from which data were extracted generally applied two‐sided statistical tests with an a priori significance level of *α* = 0.05. The design of this narrative review and reporting of statistical data adhere to standard references such as Kirkwood and Sterne (2010) and SAMPLE guideline for transparent statistical reporting. All statistical results, including effect sizes, confidence intervals, *p* values, and types of statistical tests (e.g., *χ* ^2^ test, Log‐rank test, Cox regression) reported in the Section 6, are directly extracted from the cited original publications. The referenced studies followed standard statistical reporting guidelines, with clear definitions of terms, pre‐specified versus exploratory analyses, and appropriate significance levels. Our role was to synthesize these findings qualitatively to provide a comprehensive overview of the effects of combination therapy with sorafenib in AML [18, 19]. The reviewed studies utilized various standard statistical software packages, including SPSS, SAS, R, among others, as reported. No new statistical software or analyses were performed within this review.

### 2.2 Statistical Terms and Abbreviations

The following abbreviations and statistical terms are used throughout the manuscript:

AML: acute myeloid leukemia

CR: complete remission

Cri: complete remission with incomplete hematologic recovery

PR: partial remission

EFS: event‐free survival

FLT3: fms‐like tyrosine kinase 3

FLT3‐ITD: internal tandem duplication mutation in FLT3 gene

FLT3‐TKD: tyrosine kinase domain mutation in FLT3 gene

HR: hazard ratio

ORR: overall response rate

OS: overall survival

*p* value: probability value

CI: confidence interval

SPSS: statistical package for the social sciences

FDA: food and drug administration

### 2.3 Statistical Tests

The statistical tests reported in this narrative review were performed by the original authors of the cited studies. These included *χ* ^2^ tests for categorical variables, Log‐rank tests for survival comparisons, Cox proportional hazards regression for multivariate survival analysis, Fisher's exact test for small sample categorical data, and other appropriate tests as indicated in each original publication. No new statistical tests were conducted by the authors of this review. All extracted statistical results including effect sizes, confidence intervals, and *p* values have been reported according to the original studies.

## 3 Results

### 3.1 **T**he Combination of Sorafenib With Cytarabine and Idarubicin

Cytarabine is an antimetabolite and antineoplastic agent under the anthracycline medication class. ARA‐C, also known as arabinosylcytosine, is another name for this pyrimidine analog. Within the cell, it is changed into the triphosphate form and fights with cytidine to join the DNA [20]. Idarubicin is a 4‐dimethoxy‐anthracycline derivative of daunorubicin that exhibits more activity than daunorubicin at this level by stabilizing the cleavable complex between DNA and topoisomerase II, which is how it causes cytotoxicity [21, 22].

An initial cycle of idarubicin and cytarabine followed by a second cycle of amsacrine and high‐dose cytarabine comprised standard induction therapy [23]. In the study by Yalniz et al., patients were divided into two groups: group one received induction regimens containing idarubicin and cytarabine (IA), while group two received the same regimen with the addition of sorafenib. The rates of complete remission (CR) or CR with incomplete hematologic recovery (CRi) were 85% in group one and 99% in group two, with a statistically significant difference (*p *= 0.004, *χ* ^2^ test). Median relapse‐free survival (RFS) was 12 months in group one compared to 45 months in group two, as analyzed by the log‐rank test (*p *= 0.02). These results indicate that adding sorafenib to chemotherapy not only improves outcomes in FLT3‐ITD mutant AML patients regardless of allele burden but also overcomes the adverse prognostic impact associated with higher allele burden [24]. In the study by Ravandi et al., 61 AML patients received sorafenib in addition to standard induction chemotherapy with cytarabine and idarubicin administered from Days 1–7. A response rate of 93% was observed among patients with FLT3‐mutated AML. Based on these preliminary results, sorafenib appears to provide effective disease control in the short term. However, to achieve a synergistic effect between sorafenib and cytotoxic agents, it is recommended that combination regimens incorporating sorafenib and chemotherapy be initiated early in the disease course [25]. Another study reported that induction chemotherapy with anthracyclines, including idarubicin and cytarabine, administered without sorafenib, was associated with lower CR rates (approximately 50%–60%) and higher early mortality rates, reaching up to 20% within the first 30 days posttreatment [26, 27, 28]. In a phase 2 trial, sorafenib, idarubicin, and cytarabine were administered to 18 patients with newly diagnosed AML with FLT3‐ITD. Of these patients, 17 (94%) experienced morphological CR/complete remission with inadequate platelet recovery [17]. Eventually, patients with recently identified AML, especially those with FLT3‐ITD, respond well to a regimen consisting of sorafenib, idarubicin, and cytarabine. Additionally, when used as frontline therapy for AML patients, it is safe and has a high CR rate. The decisive action of sorafenib against ERK and FLT3 signaling is confirmed by correlation studies [29, 30].

### 3.2 The Combination of Sorafenib With Cytarabine and Daunorubicin

Daunorubicin belongs to the class of antibiotics known as anthracyclines. The anti‐neoplastic effects are achieved by various pathways, including anti‐cytotoxic and anti‐mitotic actions. It inhibits the production of DNA and RNA by intercalating between DNA base pairs, which causes the DNA double helix to uncoil and inhibits the topoisomerase II enzyme, which leads to single and double‐strand breaks. Moreover, daunorubicin suppresses the activity of the polymerase enzyme, dysregulating gene expression, damaging DNA with free radicals, and ultimately causing apoptosis, mitochondrial damage, and programmed cell death.

In the study by Uy et al., 54 patients with AML were treated with sorafenib added to induction and consolidation chemotherapy consisting of daunorubicin and cytarabine, followed by a 12‐month maintenance therapy regimen. Among the participants, 39 (71%) harbored FLT3‐ITD mutations, and 15 (29%) had FLT3‐TKD mutations. For the FLT3‐ITD cohort, the 1‐year OS was 62% (95% CI, 45%–78%), which was significantly higher than the 30% OS observed in a historical control group (*χ* ^2^ test, *p* < 0.001). The FLT3‐TKD group demonstrated a 1‐year OS of 71% (95% CI, 42%–92%). Median disease‐free survival (DFS) and OS in the FLT3‐ITD patients were 12.2 months (95% CI, 5.0–16.9) and 15.0 months (95% CI, 10.4–20.1), respectively, as analyzed by the log‐rank test. In comparison, the FLT3‐TKD cohort had a median OS of 16.2 months (95% CI, 5.0 to not available [NA]) and median DFS of 9.6 months (95% CI, 1.9 to NA). These results indicate that adding sorafenib to standard chemotherapy regimens including daunorubicin and cytarabine significantly improves survival outcomes among older adults with FLT3‐mutated AML. In addition to efficacy outcomes, toxicity assessment in this elderly population was crucial. The combination of sorafenib with standard chemotherapy was generally tolerable; however, notable hematologic toxicities, including severe neutropenia and thrombocytopenia, as well as non‐hematologic adverse events such as hepatic dysfunction and skin rashes, were observed. These toxicities require careful monitoring and supportive management. Therefore, balancing treatment efficacy and toxicity is essential in the management of elderly AML patients [31]. In an additional investigation by Loo et al., an accelerated regimen of sorafenib from Days 10–19 in conjunction with daunorubicin and cytarabine enhanced event‐free survival (EFS). It was linked to an induction death rate of 3%, as opposed to 2% in the placebo‐controlled arm, in younger patients with newly diagnosed AML (including wild‐type FLT3) [32]. These findings support our conclusion that sorafenib can be added to cytarabine and daunorubicin to treat FLT3‐mutated AML. This combination has been shown to significantly prolong EFS and enhance patient survival.

### 3.3 The Combination of Sorafenib With Vorinostat and Bortezomib

Vorinostat is a histone deacetylase (HDAC) inhibitor, structurally belonging to the hydroxymate group. Both class I and class II HDAC enzymes are inhibited by vorinostat, a broad inhibitor of HDAC activity. Histones and other acetylated proteins accumulate after binding to the HDAC enzyme, which has a variety of physiological consequences. Both transcriptional and non‐transcriptional effects are observed. These medications cause abnormal homeostasis and the formation of tumors. HDAC inhibitors affect different cell types in vivo and in vitro, causing malignant cells to undergo complete apoptosis, halting proliferation, and altering cell differentiation. These medications can be taken alone or in conjunction with other anti‐neoplastic medications [33]. The clinical trial conducted by Sayar et al. included a total of 15 patients with AML who received oral sorafenib at a dose of 400 mg twice daily. Vorinostat, a histone deacetylase inhibitor, was administered orally following a 3 × 3 cohort design with escalating doses of 200, 300, and 400 mg per day divided into two doses. Thirteen patients were evaluable for treatment response. After the first treatment cycle, six patients (46%) achieved partial PR and one patient (8%) achieved CR. Notably, all patients exhibited complete clearance of peripheral blood blasts within 1 week, despite baseline blast counts ranging from 52,000 to 84,000 cells/μL. After two treatment cycles, one patient demonstrated a marked reduction in bone marrow blast percentage from 90% to 10%. Furthermore, the study suggested that the addition of bortezomib, a proteasome inhibitor known to induce endoplasmic reticulum stress, significantly enhances treatment responses. These findings support the potential of this combinational strategy for improving outcomes in refractory or advanced AML [34]. In the phase I portion of the trial by Zaid et al., 17 patients were enrolled. Patients received intravenous sorafenib at 400 mg twice daily and vorinostat at 200 mg twice daily (both for 14 days), along with bortezomib at 1.3 mg/m² on Days 1, 4, 8, and 11 of a 21‐day cycle. Among these patients, 6 (40%) achieved a clinical response, including 4 (27%) who attained CR. Notably, all responders had relapsed or refractory disease [35].

In a separate phase I study conducted by Sayar et al., 15 patients received oral sorafenib at 400 mg twice daily, alongside oral vorinostat with dose escalation in subsequent cohorts. Among these patients, 44% exhibited partial PR, and one patient (6%) achieved a complete remission lasting 5 months without evidence of disease.

The phase II portion of the Sayar et al. study included 20 patients. Vorinostat was administered orally at 200 mg twice daily, initially intermittently on Days 1–4 and 8–12, and subsequently continuously. Oral sorafenib and intravenous bortezomib were administered in successive cohorts with dose escalation. In this phase, 5 patients experienced partial platelet recovery (CRi), 2 patients (14%) attained PR, 1 patient (7%) had CRi, and 1 patient (7%) achieved CR. Combining data from all 37 patients across both phases, the overall response rates were as follows: 1 complete remission (3%), 6 CRi (16%), and 2 partial remissions (5%). All responders harbored FLT3‐ITD mutations. Despite these responses, all responders experienced relapse within two consolidation cycles, and none proceeded to transplantation due to various reasons [36].

### 3.4 The Combination of Sorafenib With Clofarabine, Fludarabine and Busulfan

In December 2004, the FDA approved clofarabine, a purine nucleoside antimetabolite, for treating pediatric patients with acute lymphoblastic leukemia that had relapsed or was refractory. Deoxycytidine kinase transforms clofarabine intracellularly into the 5′‐monophosphate metabolite, which is subsequently transformed into the active 5′‐triphosphate form by mono‐ and diphosphokinases. Clofarabine triphosphate is cytotoxic to cancer cell types quiescent or quickly growing in vitro. It inhibits DNA synthesis by acting on ribonucleotide reductase and DNA polymerases [37].

Antineoplastic drugs such as fludarabine have been explored in patients with various lymphoproliferative cancers. The active metabolite F‐ara‐A (9‐β‐d‐arabino‐furanosyl‐2‐fluoroadenine) triphosphate (F‐ara‐ATP) is thought to be responsible for stopping DNA and RNA synthesis and elongating nucleic acid chains. Other mechanisms for fludarabine's antitumor activity include inhibiting DNA and RNA polymerases, DNA primase, DNA ligase, and ribonucleotide reductase, as well as increasing the activity of deoxycytidine kinase. Apoptosis has been identified as an additional significant mechanism of fludarabine‐induced cell death in both in vitro and in vivo studies. Against human leukemia cell lines, fludarabine showed concentration‐time‐dependent cytotoxicity in vitro. It has been demonstrated that fludarabine increases the effectiveness of many anticancer drugs in vitro [38].

The chemical name for busulfan, a bifunctional alkylating agent, is 1,4‐butanediol dimethanesulfonate. Busulfan functions similarly to other alkylating drugs. The primary molecular process responsible for biological activity is the alkylation of intracellular nucleophiles. Proteins and, more crucially, nucleic acids are alkylated upon the release of the methyl sulfonate groups. This occurs mainly at the N position of guanine but also at other DNA locations. As a result, during the repair process, single‐strand breakage and incorrect reading of the DNA code are possible. Combining busulfan with various chemotherapeutic medications has been demonstrated to be a successful pretreatment plan for patients with leukemia, solid tumors, hemoglobinopathies, and immune system defects undergoing autologous or allogeneic bone marrow transplantation (BMT) [39].

Few studies were conducted in this field Andersson et al. investigated the synergistic effects of combining sorafenib with clofarabine, fludarabine, and busulfan in FLT3‐ITD positive AML. They demonstrated that adding sorafenib to the [Bu+Clo+Flu] regimen significantly enhanced cytotoxicity, leading to increased apoptosis and inhibition of cell proliferation in AML cell lines MV‐4‐11 and MOLM‐13. Mechanistically, this combination activated DNA damage response pathways, inhibited pro‐survival kinases (FLT3, MEK, AKT), increased proapoptotic proteins (such as PUMA), decreased antiapoptotic proteins (like MCL‐1), and promoted mitochondrial apoptotic signaling, including cytochrome c release and mitochondrial membrane potential reduction. Functionally, the addition of sorafenib improved the suppression of cell proliferation from approximately 20% with the triple combination to around 60% with the quadruple combination, and apoptosis induction increased markedly to about 50%. These findings support the role of sorafenib as an effective adjunct to conventional chemotherapy to enhance anti‐leukemic activity in FLT3‐ITD positive AML [40].

In a study by Popat et al., sorafenib was combined with a fractionated busulfan (f‐Bu) and fludarabine regimen in 24 patients with AML. Among these patients, 16 (66.6%) achieved CR, 5 (20.8%) achieved complete remission with incomplete hematologic recovery (CRi), and 3 (12.5%) experienced disease progression. Cytogenetic analysis revealed that 10 patients (41.7%) had an adverse‐risk karyotype. Minimal residual disease (MRD) was detectable in 13 cases (54.2%), while 9 patients (38%) harbored FLT3 mutations. With a median follow‐up of 7.6 months among the 20 surviving patients, the estimated 1‐year PFS was 89% (95% CI 75%–100%) as calculated by the Kaplan–Meier method and compared using the log‐rank test [41]. These findings indicate that the addition of sorafenib to the f‐Bu and fludarabine conditioning regimen is associated with high remission rates and favorable short‐term progression‐free survival in AML patients, including those with adverse cytogenetic profiles and FLT3 mutations.

In conclusion, in two FLT3‐ITD positive AML cell lines as well as in FLT3‐ITD positive patient‐derived leukemia cells, the multikinase inhibitor sorafenib synergistically increases the cytotoxicity achieved with nucleoside analogs and the DNA alkylating agent Bu, but not in cells that only have wild‐type FLT3. Initial efficacy findings, with 89% PFS, appear favorable, according to studies. Consequently, for AML patients with the FLT3‐ITD mutation, sorafenib in conjunction with [Bu+Clo+Flu] can be administered in a pre‐transplant conditioning regimen.

### 3.5 The Combination of Sorafenib With 5‐Azacytidine

The main difference between 5‐azacytidine and the naturally occurring pyrimidine nucleoside cytidine is that 5‐azacytidine has nitrogen instead of a fifth carbon [42]. 5‐Azacytidine has been shown to have two primary antineoplastic action mechanisms: it can directly bind to RNA, disrupting RNA metabolism in the process, and it can also prevent DNA methylation [43].

George et al. evaluated the combined effects of sorafenib and azacitidine in elderly patients with relapsed or high‐risk AML harboring FLT3‐ITD mutations. Males comprised 34% of the cohort, while 24% (*n* = 15) had secondary AML and 11% (*n* = 7) had therapy‐related AML. The median FLT3‐ITD allelic burden was 0.73 (range, 0.34–0.93). Among all patients, 8 (14%) achieved CR, 7 (13%) achieved CR without platelet recovery (CRp), and 10 (18%) attained CR without peripheral blood count recovery (CRi). One patient (1%) achieved PR. The median time from diagnosis to initiation of sorafenib plus azacitidine treatment was 5.5 months (range, 3 days to 27 months). The overall response rate (ORR) was 46%. OS was 45% at 3 months and decreased to 24% at 12 months. The FLT3‐ITD allelic burden did not significantly impact OS (hazard ratio [HR], 0.93; 95% CI, 0.79–1.10; Cox proportional hazards model, *p *= 0.433). The only variable significantly associated with ORR was the percentage of blasts (odds ratio [OR], 1.025; 95% CI, 1.007–1.043; logistic regression, *p *= 0.007). The ORR was not significantly influenced by the FLT3‐ITD allelic burden (OR, 0.63; 95% CI, 0.21–1.83; logistic regression, *p *= 0.394) [44]. These findings indicate that while the combined sorafenib and azacitidine treatment yields an overall response rate of nearly 50% in this elderly, high‐risk AML population, the FLT3‐ITD allelic burden did not significantly predict survival or response outcomes. Instead, disease burden as measured by blast percentage was a significant predictor of treatment response. Ohanian et al. conducted a study including 27 newly diagnosed, treatment‐naïve patients with FLT3‐mutated AML who received first‐line therapy with a combination of 5‐azacytidine (AZA) and sorafenib. The overall response rate was 78%, comprising CR, complete remission with incomplete count recovery (CRi/CRp), and PR. Patients received a median of three treatment cycles. The median duration of remission (CR, CRi, and CRp) was approximately 14.5 months (range: 1.1–28.7 months). Among responders, three patients (one CR and two CRi) proceeded to allogeneic stem cell transplantation. For surviving patients, the median follow‐up was 4.1 months (range: 3.0–17.3 months). Median OS was 8.3 months for the entire cohort and 9.2 months for the 19 responders. The treatment regimen was well tolerated in this elderly, treatment‐naïve FLT3‐mutated AML population, with no premature deaths reported [45].

Campregher et al. investigated a 49‐year‐old man with de novo AML who was first treated with one cycle of 7 + 3. He achieved morphologic complete remission with minimal residual disease (0.1%) as determined by flow cytometry. Sorafenib was begun immediately following donor lymphocyte infusion (DLI) at 400 mg twice daily. Azacytidine therapy began 1 month after DLI at a dose of 32 mg/m^2^ every 5 days, once a month. After 28 months of DLI, the patient was still in complete remission with 100% donor chimerism, according to the most recent bone marrow evaluation [46]. Eventually, the Sorafenib plus Azacitidine combination is well tolerated and highly effective in treating relapsed or refractory AML with FLT3‐ITD.

### 3.6 The Combination of Sorafenib With Quizartinib, Midostaurin, and Giltertinib

As a potent and selective FLT3 inhibitor for AML, Quizartinib (AC220) is a potent and selective second‐generation class III receptor TKI, highly effective against FLT3‐ITD mutated AML cells has been primarily evaluated in relapsed and refractory AML settings [47, 48].

Midostaurin is a first‐in‐class FLT3 inhibitor, initially discovered as a protein kinase C inhibitor derived from *Streptomyces staurosporeus*. Its FLT3‐inhibitory effect reduces autophosphorylation of FLT3 and downstream signaling via p38 MAPK and STAT5 pathways, inducing apoptosis in FLT3‐ITD‐expressing cells. Midostaurin has been approved for treating newly diagnosed FLT3‐mutated AML patients, typically in combination with standard chemotherapy. Moreover, its inhibitory effects on kinases such as SYK and KIT may contribute to a chemosensitizing effect even in FLT3 wild‐type patients [49, 50].

Gilteritinib is a second‐generation multi‐kinase inhibitor that targets FLT3 ITD and TKD mutations (e.g., FLT3‐ITD, FLT3‐ITD‐D835Y, FLT3‐D835Y), as well as other kinases including AXL, ALK, and c‐KIT. It effectively induces apoptosis in FLT3‐ITD expressing AML cells. Gilteritinib is approved for relapsed/refractory (R/R) FLT3‐mutated AML and has shown superior efficacy compared to salvage chemotherapy [30, 51].

In this context, In a study by Othman et al., 59 patients with molecular failure (52 with FLT3‐ITD, 7 with TKD, 3 both) were treated with various FLT3 inhibitors including gilteritinib (*n* = 38), quizartinib (*n* = 7), and sorafenib (*n* = 11). More than half had prior exposure to midostaurin. Molecular failure occurred at a median of 9.2 months post‐diagnosis. Among these patients, 60% achieved molecular response and 45% became MRD negative. Due to low hematologic toxicity, 22 patients proceeded to allogeneic stem cell transplantation (Allo‐SCT) without delay, and 6 received donor lymphocyte infusions. The 2‐year molecular event‐free survival and overall survival rates were 56% (95% CI: 44–72) and 80% (95% CI: 69–93) as calculated by Kaplan–Meier survival analysis. Differences in survival were likely assessed using the log‐rank test, although not explicitly reported [52].

A multicenter country‐wide study by Shimony et al. evaluated post‐transplant TKI maintenance in 41 patients receiving sorafenib (*n* = 23) or midostaurin (*n* = 18). Most patients (79%) had also received TKIs pre‐transplant. At a median follow‐up of 10 months, 71% remained alive in complete remission. Multivariate analysis identified both TKI type (sorafenib vs. midostaurin) and a hematopoietic cell transplantation‐comorbidity index (HCT‐CI) score of less than 4 as significant predictors of longer OS (statistical methods likely included Cox proportional hazards regression; exact hazard ratios and P‐values were not provided in the summary). The toxicity profile was acceptable, although 17% of patients discontinued TKI therapy due to adverse events [53].

In intensively treated patients with newly diagnosed FLT3‐ITD‐mutated AML, Sasaki et al. demonstrated that adding sorafenib to induction chemotherapy improved the response rate to 98% compared to 83% in patients receiving chemotherapy alone (*χ* ^2^ test, *p* = 0.057). EFS was significantly prolonged from a median of 8 months to 35 months (log‐rank test, *p* = 0.019), and OS increased from 13 months to 42 months (log‐rank test, *p* = 0.026). Multivariate Cox proportional hazards regression analysis identified sorafenib as an independent favorable prognostic factor for survival outcomes [54].

Furthermore, based on its demonstrated better response and survival outcomes when compared with salvage chemotherapy (SC), the FLT3 inhibitor Gilteritinib was recommended for relapsed or refractory FLT3‐mutated AML in the Perl et al. trial. In relapsed or refractory (R/R) FLT3‐mutated AML, gilteritinib demonstrated high composite complete remission (CRc) rates in both patients previously treated with FLT3 TKIs, such as midostaurin or sorafenib, and in TKI‐naïve patients. Analysis of the CHRYSALIS and ADMIRAL trials showed that 42%–52% of patients had prior FLT3 TKI exposure. Notably, median OS and CRc rates were superior in the gilteritinib arm compared to salvage chemotherapy, even among patients with prior TKI treatment. Specifically, in the ADMIRAL trial, the CRc rate among patients with prior FLT3 inhibitor exposure was 52%, with a HR for OS of 0.60 (95% CI, 0.30–1.21), indicating a favorable trend toward improved survival. These survival differences were likely assessed using the log‐rank test, and the HR estimated via Cox proportional hazards regression. However, remission duration was typically shorter among patients previously exposed to FLT3 inhibitors, reflecting more resistant disease biology [55].

In conclusion, Midostaurin was found to work synergistically with standard chemotherapeutic medications and some targeted treatments against AML cells that did not have FLT3 mutations. This process could be aided by midostaurin's distinct kinase profile, which includes proteins essential in AML transformation such as SYK and KIT, and suppression of the ERK pathway or promiscuous signaling. Midostaurin appears to be a chemosensitizing drug in AML patients who do not have the FLT3 mutation [56].

## 4 Conclusion

Table 1 shows a summary of the factors described in this review. Sorafenib is commonly used in AML treatment, however, its effectiveness as monotherapy is limited and thus combination therapies are being explored. The combination of sorafenib with chemotherapeutic agents such as antimetabolites, FLT3 inhibitors, proteasome inhibitors, alkylating agents, HDAC inhibitors, and topoisomerase II inhibitors seems promising based on several studies and can enhance its antitumor activity. It should be noted that the positive effects of combination therapies containing sorafenib are not limited to patients with FLT3 mutations. This drug, through multi‐targeted inhibition of signaling pathways, enhances the sensitivity of AML cells to chemotherapy even in patients without FLT3 mutations and improves treatment response.

**Table 1:** Combinations of the chemotherapy drugs with sorafenib are mainly described in this review.

Design of the study	Results	Ref
**Sorafenib in combination with Cytarabine and Idarubicin**		
Patients were divided into two groups: those who got regimens containing induction of idarubicin and cytarabine (IA, group one) and those who also received regimens containing induction of sorafenib (group two).	For both groups, the percentages of complete remission (CR)/CR with incomplete hematologic recovery (CRi) were 85% and 99%, respectively (*p* = 0.004).	[22]
From Day 1 to Day 7, 61 AML patients received sorafenib in addition to traditional induction chemotherapy, which included idarubicin and cytarabine.	Demonstrated a 93% response rate in AML7 individuals with the FLT3 mutation.	[23]
Used treatment without sorafenib, mixing an anthracycline such as idarubicin with cytarabine.	Reduced complete remission (CR; 50%–60%) rate and more early mortality (up to 20% at 30 days).	[24, 25, 26]
Phase 1 clinical trials have evaluated sorafenib as a single treatment for patients with recurrent or refractory AML.	There was evidence of clinical action, as 5 individuals (10%) achieved complete remission (CR) or full hematological recovery (CRi), while 17 other patients (34%) exhibited a significant decrease in bone marrow and/or peripheral blood blasts. These patients were all found to have FLT3‐ITD mutations.	[27]
Cytarabine and idarubicin have also been used with sorafenib. This regimen was used to treat 18 newly diagnosed AML patients with FLT3‐ITD in a phase 2 study.	A morphological complete remission or CR was reached by 17 of the 18 patients (94%) with inadequate platelet recovery.	[17]
**Sorafenib in combination with Cytarabine and Daunorubicin**		
Sorafenib was added to daunorubicin and cytarabine‐based induction and consolidation chemotherapy and was also continued for 12 months of maintenance therapy.	For FLT3‐ITD patients, the observed 1‐year OS ranged from 45% to 78%, while for FLT3‐TKD patients, it was 71% (42% to 92%). For the FLT3‐TKD group, the median OS and disease‐free survival were 9.6 and 16.2 months, respectively, whereas for the FLT3‐ITD group, they were 12.2 and 15.0 months, respectively.	[33]
For patients with recently diagnosed AML (including wild‐type FLT3), an accelerated regimen of sorafenib in combination with daunorubicin and cytarabine from Days 10–19.	Correlated with a 3% induction death rate, as opposed to 2% in the placebo‐controlled arm, and enhanced event‐free survival (EFS).	[34]
**Sorafenib in combination with Vorinostat and Bortezomib**		
A total of 15 patients were included, and oral sorafenib 400 mg bid and oral vorinostat ascending doses were provided in a 3 × 3 cohort design.	Thirteen of the 15 treated individuals could be evaluated for response. After the first treatment cycle, 6 out of 13 patients (46%) showed PR, while 1 out of 13 patients (8%) showed CR after just one cycle. Each had a total clearance of peripheral blood blasts in one week, from as high as 52–84,000/µL; after two cycles, one had marrow blast decrease to 10% (from an initial 90%).	[37]
In the phase I portion, 17 patients were recruited. Every 21 days on Days 1, 4, 8, and 11, patients were given sorafenib 400 mg bid, vorinostat 200 mg bid (both for 14 days), and bortezomib 1.3 mg/m^2^ IV.	Six patients (40%) had a response, and 4 (27%) had a full remission.	[38]
In a phase I trial, 15 patients were recruited and given 400 mg twice daily of sorafenib orally, with dose escalation of oral vorinostat administered in subsequent cohorts.	Of the patients, 44% showed signs of partial remission (PR), and 6% had a complete remission (CR) lasting five months without experiencing any disease.	[39]
There were 20 participants recruited in the phase II research. 200 mg of vorinostat was administered orally twice a day, at first only intermittently on Days 1–4 and 8–12, and then constantly. The administration of oral sorafenib and intravenous borte zomib was done in successive cohorts with dose escalation.	Five patients had partial recovery of their platelets (CRi). Two patients (14%) had a PR, 1 patient (7%) had CRi, and 1 patient (7%) had CR.	[39]
**Sorafenib in combination with Clofarabine and Fludarabine and Busulfan**		
Researchers looked at the synergism between clofarabine (Clo), fludarabine (Flu), busulfan (Bu), and sorafenib (Sor) to further improve their efficacy in FLT3‐ITD positive AML.	When FLT3‐ITD positive MV‐4‐11 and MOLM 13 cells were exposed to [Bu+Clo+Flu+Sor], it produced synergistic cytotoxicity. When [Bu+Clo+Flu] was combined, it produced a ~ 20% reduction in cell proliferation. When Sor was added to the [Bu+Clo+Flu] mixture, the inhibition of growth was increased to around 60%. Apoptosis was not triggered in cells exposed to Bu, Clo, Flu, or Sor alone. However, 5%–10% of cells exposed to two or three drug combinations [Bu+Clo], [Bu+Flu], [Clo+Flu], and [Bu+Clo+Flu] experienced apoptosis. When Sor was added to the [Bu+Clo+Flu] mixture, apoptosis was greatly increased, reaching about 50%. Exposure to Sor alone did not reduce the potential of the mitochondrial membrane; however, exposure to [Bu+Clo+Flu] and [Bu+Clo+Flu+Sor] considerably reduced the potential of the mitochondrial membrane by around 20% and 55%, respectively.	[44]
They added sorafenib to fludarabine and fractionated busulfan regimen (f‐bu)	Of the 24 patients, 16 (66.6%) had CR disease, 5 (20.8%) had CRi illness, and 3 (12.5%) had advanced disease. A karyotype indicative of adverse risk was found in 10 (41.7%) subjects. There was MRD in 13 (54.2%) cases. Nine (38%) had flt3 mutations. In 20 surviving patients, the median follow‐up was 7.6 months, and 89% (95% CI 75%–100%) of them were 1‐year progression‐free.	[45]
**Sorafenib in combination with 5‐Azacytidine**		
Examining the synergistic effects of azacitidine and sorafenib in elderly relapsed or at‐risk patients with AML mutant for FLT3‐ITD.	One patient (1%) experienced a partial response (PR), whereas 8 patients (14%) experienced a complete response (CR), with 7 patients (13%) obtaining CR without platelet recovery (CRp) and 10 patients (18%) achieving CR without peripheral blood count recovery (CRi). 46% of respondents responded overall. The OS was 24% at 12 months and 45% at 3 months. The overall survival (OS) was not significantly affected by the FLT‐3 ITD allele load.	[48]
Examined the use of 5‐azacytidine (AZA) with sorafenib in patients with FLT3‐mutated acute myeloid leukemia (AML) who had relapsed or were not responding to treatment. We examined 27 recently diagnosed patients with untreated FLT3‐mutant AML who were started on first‐line therapy using two different regimens of AZA plus sorafenib.	CR/CRp/CRi has an average length of 14.5 months (1.1–28.7 months). Three patients (11%) who responded (1 CR, 2 CRi) received allogeneic stem cell transplantation. For patients who survived, the average follow‐up period was 4.1 months (3.0–17.3 months). For the total cohort, the median overall survival was 8.3 months, whereas for the 19 responders, it was 9.2 months.	[49]
The first course of treatment for a 49‐year‐old male patient with de novo AML was one cycle of 7 + 3, and sorafenib (400 mg twice daily) was begun right away following donor lymphocyte infusion (DLI). One month following DLI, azacytidine therapy was initiated at a dose of 32 mg/m^2^ every five days, once a month.	After 28 months of DLI, the patient' most recent bone marrow evaluation revealed that they were still in full remission with 100% donor chimerism.	[50]
For 7 days, 40 patients with FLT3/ITD mutation received intravenous administration of AZA 75 mg/m^2^/d and oral administration of sorafenib 400 mg continuously.	The complete remission with incomplete count recovery (CRi) was 27%, the full remission (CR) was 16%, and the partial remission (PR) was 3%. The response rate (RR) was 46%.	[51]
Intravenously administered AZA 75 mg/m^2^/d for 7 days and orally administered sorafenib 400 mg continuously in the pregnant patient.	Successfully treated a pregnant patient with FLT3/ITD‐mutated AML.	[52]
**Sorafenib in combination with Quizartinib, Midostaurin and Giltertinib**		
The study included 59 patients. More than half of the patients have gotten midostaurin in the past. Molecular failure was treated with gilteritinib (*n* = 38), quizartinib (*n* = 7), or sorafenib (*n* = 11) and occurred at a median of 9.2 months after diagnosis.	A molecular reaction was obtained by 60%, and 45% of them reached MRD negative. Because of the low hematological toxicity, 22 patients underwent an allogeneic transplant without delay, and six more underwent donor lymphocyte infusion. The 2‐year survival rate was 80% (95%CI 69–93) and 56% (95%CI 44–72) for molecular event‐free survival.	[52]
Evaluated 41 patients on post‐transplant TKIs (midostaurin, n ¼ 18; sorafenib, n ¼ 23). Pre‐transplant TKIs were also administered to most (n ¼ 32, 79%).	Upon a follow‐up of 10 months post‐transplant (range 3–53.6), 29 patients (71%) remained alive and in full remission.	[53]
In a study, the clinical outcomes of patients with relapsed or refractory FLT3‐mutated AML in the CHRYSALIS and ADMIRAL trials were retrospectively compared to those of patients who had not previously received FLT3 TKI exposure and had received midostaurin or sorafenib.	Patients who had previously had a FLT3 TKI before gilteritinib (CHRYSALIS, 42%; ADMIRAL, 52%) and those who had not previously received a FLT3 TKI therapy (CHRYSALIS, 43%; ADMIRAL, 55%) both showed comparable high rates of composite complete remission (CRc). Patients who had previously received an FLT3 TKI in ADMIRAL showed a longer median overall survival (HR = 0.602; 95% CI: 0.299, 1.210) and a greater CRc rate (52%) in the gilteritinib arm compared to the SC arm.	[55]

## Author Contributions

**Mobina Nakhaei Shamahmood:** conceptualization, investigation, writing – original draft, writing – review and editing. **Abolfazl Miri:** methodology, writing – original draft. **Fatemeh Mezginejad:** conceptualization, investigation, writing – review and editing, supervision, formal analysis, validation, and project administration.

## Conflicts of Interest

The authors declare no conflicts of interest.

## Transparency Statement

The corresponding author, Fatemeh Mezginejad, affirms that this manuscript is an honest, accurate, and transparent account of the study being reported; that no important aspects of the study have been omitted; and that any discrepancies from the study as planned (and, if relevant, registered) have been explained.

## Data Availability

The authors confirm that the data supporting the findings of this study are available within the article and its cited references.
